# Complete genome sequences of eight *Pasteurella multocida* isolates representing all lipopolysaccharide outer core loci

**DOI:** 10.1128/mra.00604-24

**Published:** 2024-10-04

**Authors:** Amro Hashish, Timothy J. Johnson, Mostafa Ghanem, Yuko Sato, Nubia R. Macedo, Karen J. LeCount, Mohamed El-Gazzar

**Affiliations:** 1 Department of Veterinary Diagnostic and Production Animal Medicine, College of Veterinary Medicine, Iowa State University, Ames, Iowa, USA; 2 National Laboratory for Veterinary Quality Control on Poultry Production, Animal Health Research Institute, Agriculture Research Center, Giza, Egypt; 3 Department of Veterinary and Biomedical Sciences, College of Veterinary Medicine, University of Minnesota, Minneapolis, Minnesota, USA; 4 Department of Veterinary Medicine, Virginia-Maryland College of Veterinary Medicine, University of Maryland, College Park, Maryland, USA; 5 Diagnostic Bacteriology & Pathology Laboratory, National Veterinary Services Laboratories, U.S. Department of Agriculture, Animal and Plant Health Inspection Service, Veterinary Service, Ames, Iowa, USA; Portland State University, Portland, Oregon, USA

**Keywords:** *Pasteurella multocida*, Heddleston serotyping, somatic serotypes, lipopolysaccharide, complete genome sequence, hybrid assembly

## Abstract

*Pasteurella multocida* (PM) is a major bacterial pathogen that causes fowl cholera disease in both domestic poultry and wild birds. Here, we report the complete genome sequences of eight PM isolates representing all known lipopolysaccharide outer core loci, which are phenotypically expressed as 16 known PM serotypes.

## ANNOUNCEMENT

Fowl cholera (FC), a disease caused by *Pasteurella multocida* (PM)*,* is a contagious septicemic disease of domestic and wild birds associated with high morbidity and mortality (1). Industry reports continue to list FC as one of the top health problems the broiler, layer, and turkey industries are still facing (2).

PM isolates are differentiated into 16 somatic [lipopolysaccharide (LPS)] serotypes using Heddleston gel diffusion precipitin test (3, 4). However, Heddleston serotyping has several limitations, including poor reproducibility, the inability to type many isolates, and cross-reactivity between serotypes (4–6). Analysis of the LPS structure was conducted and showed that only eight unique LPS (L1–L8) outer core biosynthesis loci are found in the 16 Heddleston types (4). Subsequently, a multiplex PCR was developed to replace the Heddleston serotyping and type PM strains into one of the eight distinct LPS genotypes (4). Here, we report the complete genome sequences and annotation of eight PM isolates representing all eight LPS outer core biosynthesis loci. The availability of these genome sequences will promote the understanding of different PM LPS genotypes and offer new insight into prevention and control strategies of FC in all bird species.

Eight PM isolates were obtained from the Bacteriology Lab in the National Veterinary Services Laboratory. Bacterial isolation was performed from saved semi-solids (BD Diagnostic Systems, Sparks, MD, USA) of diagnostic and reference cultures. The cultures were plated onto blood agar (Remel, Lenexa, KS, USA) so that a single isolated colony could be picked and inoculated into fresh media. Then, bacterial DNA was extracted using MagMax (7). Both Illumina and Nanopore sequencing were conducted similar to our previous report (8). Extracted DNA was used for the preparation of sequencing libraries using the Illumina DNA Prep Tagmentation Kit (previously called Nextera DNA Flex) with IDT for Illumina DNA/RNA UD indexes and set according to the manufacturer’s protocol (Illumina, USA). Sequencing was performed using Illumina MiSeq system (v3 reagent kit using 2 × 300 bp paired-end reads). For nanopore sequencing, the Circulomics Nanobind CBB Big DNA Kit (Circulomics, Baltimore, MD, USA) was used for the extraction of high molecular weight DNA. Libraries were prepared using SQK-LSK109 and EXP-NBD104 kits according to the manufacturer’s protocol (Oxford Nanopore Technologies, UK).

Short reads generated using Illumina were trimmed for quality and sequencing adapters using Trimmomatic version 0.33, using default parameters (9). NanoFilt version 2.8.0 was used with default parameters to quality filter Nanopore reads and filter for sequences less than 1,000 bp in length (10). Hybrid assemblies were performed using Unicycler version 0.5.0 using default parameters (11). The presence or absence of plasmid from the genome sequences was determined using the Plasmid database (12) available via PLSDB https://ccb-microbe.cs.uni-saarland.de/plsdb/.

All of the genomes were annotated using the Pathosystems Resource Integration Center (PATRIC) (13) genome annotation tool, which adopts the RAST tool kit (RASTtk) (14).

A summary of the data associated with the eight sequenced PM strains, reads generated by each sequencing platform, assembly statistics, annotation of the closed genomes, sequence typing, average nucleotide identity, and GenBank accession numbers are presented in Table 1.

**TABLE 1:** Data associated with the eight sequenced PM strains, reads generated by each sequencing platform, assembly statistics, annotation of the closed genomes, sequence typing, average nucleotide identity, and GenBank accession numbers

Parameter		Data							
Metadata		PM-10159	PM-1702	PM-10957	PM-2192	PM-2095	PM-2237	PM-1581	PM-2723
	Host	Turkey	Turkey	Turkey	Turkey	Turkey	Turkey	Pine Siskin	Turkey
	Geographical location	USA-Minnesota	USA-Virginia	USA-Arkansas	USA-Texas	USA-Minnesota	USA-Iowa	USA- Massachusetts	USA-Indiana
	Date of sample collection	3/3/2015	Unknown	8/28/2019	Unknown	Unknown	Unknown	Unknown	Unknown
Raw sequence reads	Illumina paired-end read length (nt)	300	250	300	250	250	250	250	250
	Number of Illumina reads used	244,732	973,588	210,138	868,126	1,056,934	1,437,582	1,319,566	1,393,248
	Average Illumina coverage	32×	104×	27×	96×	113×	151×	146×	152×
	Number of Nanopore reads	2,300	29,033	19,162	58,979	15,947	29,000	27,322	16,492
	Average Nanopore coverage	14×	176×	147×	350×	94×	161×	192×	108×
	Nanopore reads *N*_50_ (bp)	33,748	34,507	40,787	36,346	34,937	29,770	39,271	36,641
Assembly statistics for the closed genomes	No. of contigs	1	2	1	2	2	1	2	2
	Total length of the chromosome (bp)	2,277,732	2,337,700	2,314,161	2,268,200	2,338,462	2,379,541	2,253,720	2,297,391
	Contig matching a plasmid	Absent	Present	Absent	Present	Present	Absent	Present	Present
	Total length of the plasmid	NA	2,516	NA	3,346	3,682	NA	1,812	2,507
	GC content (%)	40.48	40.44	40.35	40.43	40.33	40.20	40.47	40.37
	*N*_50_ for the hybrid assembly (bp)	2,277,732	2,337,700	2,314,161	2,268,200	2,338,462	2,379,541	2,253,720	2,297,391
Annotation results	Number of coding sequences	2,128	2,273	2,126	2,116	2,226	2,260	2,102	2,211
	Number of tRNAs	58	56	57	57	60	55	57	57
	Number of rRNAs	19	19	19	19	19	19	19	19
	Hypothetical proteins	273	301	261	264	323	326	253	284
	Proteins with functional assignments	1,854	1,972	1,865	1,852	1,903	1,934	1,849	1,927
	Proteins with Enzyme Commission numbers assignments (15)	714	768	726	720	737	722	723	759
	Proteins with Gene Ontology assignments (16)	559	604	572	568	578	568	570	600
	Proteins with Pathway assignments (17)	496	534	508	499	513	499	505	524
	Proteins with PATRIC genus-specific family (PLfam) assignments (18)	2,101	2,232	2,081	2,058	2,182	2,217	2,030	2,155
	Proteins with PATRIC cross-genus family (PGfam) assignments (18)	2,103	2,235	2,090	2,072	2,184	2,221	2,039	2,168
DNA fingerprinting	Restriction endonuclease analysis, digestion with HhaI (19)	1,014	0005	1,023	0006	0009	0015	0008	0016
Serotyping	Using gel diffusion precipitin test (GDPT) (19)	1	5	4	6	9	15	8	16
ANIb (%)^*a*^ (cut-off threshold of 96%) (20)	To the PM type strain (NCTC10322) LT906458.1	96.28	98.47	98.39	97.99	98.31	98.17	96.08	98.42
Genotypes	Lipopolysaccharide genotype^*b*^	L1	L2	L3	L4	L5	L6	L7	L8
GenBank data	BioSample accession number	SAMN28548360	SAMN28553018	SAMN28552779	SAMN28553023	SAMN28553020	SAMN28553350	SAMN28552999	SAMN28553054
	BioProject accession number	PRJNA839711	PRJNA839711	PRJNA839711	PRJNA839711	PRJNA839711	PRJNA839711	PRJNA839711	PRJNA839711
	SRA (MinION) SRA (Illumina)	SRX15391466 SRX15391459	SRX15391475 SRX15391487	SRX15391465 SRX15391458	SRX15391477 SRX15391461	SRX15391476 SRX15391460	SRX15391479 SRX15391463	SRX15391473 SRX15391485	SRX15391478 SRX15391462
	Complete genome assembly accession number	Chromosome CP097604	Chromosome CP097616	Chromosome CP097606	Chromosome CP097620	Chromosome CP097618	Chromosome CP117196	Chromosome CP097614	Chromosome CP097622
			Plasmid CP097617		Plasmid CP097621	Plasmid CP097619		Plasmid CP097615	Plasmid CP097623

^*a*^ Average nucleotide identity percentage was calculated based on BLAST+ (21) available via JSpeciesWS online service https://jspecies.ribohost.com/jspeciesws/#home.

^*b*^ Lipopolysaccharide genotyping according to Harper et al. (4).

## Data Availability

The genomes are available from NCBI BioProject number PRJNA839711 under the accession numbers shown in Table 1.

## References

[1] Hofacre CL, Blackall PJ. 2020. Fowl cholera, p 831–846. In Swayne DE, Boulianne M, Logue CM, McDougald LR, Nair V, Suarez DL (ed), Diseases of poultry, 14th ed. Wiley‐Blackwell.

[2] Sato Y, Niel K, United States Animal Health Association. 2022. Transmissible diseases of poultry & avian species. Available from: https://usaha-org.translate.goog/transmissible-diseases-of-poultry-avian-species/?_x_tr_sl=en&_x_tr_tl=ar&_x_tr_hl=ar&_x_tr_pto=sc

[3] Heddleston KL, Gallagher JE, Rebers PA. 1972. Fowl cholera: gel diffusion precipitin test for serotyping Pasteurella multocida from avian species. Avian Dis 16:925–936. doi:10.2307/1588773 4628021

[4] Harper M, John M, Turni C, Edmunds M, St Michael F, Adler B, Blackall PJ, Cox AD, Boyce JD. 2015. Development of a rapid multiplex PCR assay to genotype Pasteurella multocida strains by use of the lipopolysaccharide outer core biosynthesis locus. J Clin Microbiol 53:477–485. doi:10.1128/JCM.02824-14 25428149 PMC4298526

[5] Singh R, Blackall PJ, Remington B, Turni C. 2013. Studies on the presence and persistence of Pasteurella multocida serovars and genotypes in fowl cholera outbreaks. Avian Pathol 42:581–585. doi:10.1080/03079457.2013.854861 24224551

[6] Wilson MA, Morgan MJ, Barger GE. 1993. Comparison of DNA fingerprinting and serotyping for identification of avian Pasteurella multocida isolates. J Clin Microbiol 31:255–259. doi:10.1128/jcm.31.2.255-259.1993 8432810 PMC262745

[7] Hashish A, Sinha A, Mekky A, Sato Y, Macedo NR, El-Gazzar M. 2021. Development and validation of two diagnostic real-time PCR (TaqMan) assays for the detection of Bordetella avium from clinical samples and comparison to the currently available real-time TaqMan PCR assay. Microorganisms 9:2232. doi:10.3390/microorganisms9112232 34835358 PMC8619015

[8] Hashish A, Johnson TJ, Chundru D, Williams ML, Sato Y, Macedo NR, Clessin A, Gantelet H, Bost C, Tornos J, Gamble A, LeCount KJ, Ghanem M, Boulinier T, El-Gazzar M. 2023. Complete genome sequences of two Pasteurella multocida isolates from seabirds. Microbiol Resour Announc 12:e0136522. doi:10.1128/mra.01365-22 36971563 PMC10112064

[9] Bolger AM, Lohse M, Usadel B. 2014. Trimmomatic: a flexible trimmer for Illumina sequence data. Bioinformatics 30:2114–2120. doi:10.1093/bioinformatics/btu170 24695404 PMC4103590

[10] De Coster W, D’Hert S, Schultz DT, Cruts M, Van Broeckhoven C. 2018. NanoPack: visualizing and processing long-read sequencing data. Bioinformatics 34:2666–2669. doi:10.1093/bioinformatics/bty149 29547981 PMC6061794

[11] Wick RR, Judd LM, Gorrie CL, Holt KE. 2017. Unicycler: resolving bacterial genome assemblies from short and long sequencing reads. PLoS Comput Biol 13:e1005595. doi:10.1371/journal.pcbi.1005595 28594827 PMC5481147

[12] Schmartz GP, Hartung A, Hirsch P, Kern F, Fehlmann T, Müller R, Keller A. 2022. PLSDB: advancing a comprehensive database of bacterial plasmids. Nucleic Acids Res 50:D273–D278. doi:10.1093/nar/gkab1111 34850116 PMC8728149

[13] Wattam AR, Abraham D, Dalay O, Disz TL, Driscoll T, Gabbard JL, Gillespie JJ, Gough R, Hix D, Kenyon R, et al.. 2014. PATRIC, the bacterial bioinformatics database and analysis resource. Nucleic Acids Res 42:D581–D591. doi:10.1093/nar/gkt1099 24225323 PMC3965095

[14] Brettin T, Davis JJ, Disz T, Edwards RA, Gerdes S, Olsen GJ, Olson R, Overbeek R, Parrello B, Pusch GD, Shukla M, Thomason JA, Stevens R, Vonstein V, Wattam AR, Xia F. 2015. RASTtk: a modular and extensible implementation of the RAST algorithm for building custom annotation pipelines and annotating batches of genomes. Sci Rep 5:8365. doi:10.1038/srep08365 25666585 PMC4322359

[15] Schomburg I, Chang A, Ebeling C, Gremse M, Heldt C, Huhn G, Schomburg D. 2004. BRENDA, the enzyme database: updates and major new developments. Nucleic Acids Res 32:D431–D433. doi:10.1093/nar/gkh081 14681450 PMC308815

[16] Ashburner M, Ball CA, Blake JA, Botstein D, Butler H, Cherry JM, Davis AP, Dolinski K, Dwight SS, Eppig JT, Harris MA, Hill DP, Issel-Tarver L, Kasarskis A, Lewis S, Matese JC, Richardson JE, Ringwald M, Rubin GM, Sherlock G. 2000. Gene ontology: tool for the unification of biology. Nat Genet 25:25–29. doi:10.1038/75556 10802651 PMC3037419

[17] Kanehisa M, Sato Y, Kawashima M, Furumichi M, Tanabe M. 2016. KEGG as a reference resource for gene and protein annotation. Nucleic Acids Res 44:D457–D462. doi:10.1093/nar/gkv1070 26476454 PMC4702792

[18] Davis JJ, Gerdes S, Olsen GJ, Olson R, Pusch GD, Shukla M, Vonstein V, Wattam AR, Yoo H. 2016. PATtyFams: protein families for the microbial genomes in the PATRIC database. Front Microbiol 7:118. doi:10.3389/fmicb.2016.00118 26903996 PMC4744870

[19] LeCount KJ, Schlater LK, Stuber T, Robbe Austerman S, Frana TS, Griffith RW, Erdman MM. 2018. Comparison of whole genome sequencing to restriction endonuclease analysis and gel diffusion precipitin-based serotyping of Pasteurella multocida. J Vet Diagn Invest 30:42–55. doi:10.1177/1040638717732371 28906178 PMC6504148

[20] Ciufo S, Kannan S, Sharma S, Badretdin A, Clark K, Turner S, Brover S, Schoch CL, Kimchi A, DiCuccio M. 2018. Using average nucleotide identity to improve taxonomic assignments in prokaryotic genomes at the NCBI. Int J Syst Evol Microbiol 68:2386–2392. doi:10.1099/ijsem.0.002809 29792589 PMC6978984

[21] Richter M, Rosselló-Móra R, Oliver Glöckner F, Peplies J. 2016. JSpeciesWS: a web server for prokaryotic species circumscription based on pairwise genome comparison. Bioinformatics 32:929–931. doi:10.1093/bioinformatics/btv681 26576653 PMC5939971

